# Correlation of Serum Levels of Vitronectin, Malondialdehyde and Hs- CRP With Disease Severity in Coronary Artery Disease

**DOI:** 10.15171/jcvtr.2015.24

**Published:** 2015

**Authors:** Alireza Yaghoubi, Morteza Ghojazadeh, Sakhavat Abolhasani, Hossein Alikhah, Fatemeh Khaki-Khatibi

**Affiliations:** ^1^ Rajaie Cardiovascular Medical and Research Center, Iran University of Medical Sciences, Tehran, Iran; ^2^ Liver and Gastrointestinal Disease Research Center, Tabriz University of Medical Sciences, Tabriz, Iran; ^3^ Students’ Research Committee, Tabriz University of Medical Sciences, Tabriz, Iran; ^4^ Department of Emergency Medicine, Tabriz University of Medical Sciences, Tabriz, Iran; ^5^ Drug Applied Research Center and Department of Clinical Biochemistry, Faculty of Medicine, Tabriz University of Medical Sciences, Tabriz, Iran

**Keywords:** Vitronectin, MDA, Hs-CRP, Correlation, CAD, Severity

## Abstract

*Introduction:* Vitronectin (VN), malondialdehyde (MDA) and high-sensitivity C-reactive rotein (hs-CRP) are cooperative agents involved in the atherosclerosis process. The study was conducted to assess the correlation of VN, MDA and hs-CRP with the severity of coronary artery disease (CAD).

*Methods:* Parameters such as serum VN, MDA and hs-CRP were measured in 250 subjects including 200 patients with angiographically diagnosed CAD (50 subjects with non-significant CAD, 50 with single vessel disease, 50 with double vessel disease, and 50 with triple vessel disease) and 50 CAD-free subjects as a control group. Serum VN was measured with ELISA; MDA was measured based on reaction with thiobarbituric acid (TBA); and hs-CRP level was measured by a Commercial Kit by Immunoturbidimetry.

*Results:* Serum VN, MDA and hs-CRP were significantly higher in patient groups compared to control group (*P* < .05). The mean value of MDA between 1 vessel and 3 vessel groups had significant difference (*P* = .01), also mean value of MDA between 2 vessel and control group and normal group showed significant difference (*P* < .001). The difference of MDA between 3 vessel and normal and 1 vessel and control group was significant (*P* < .001).

*Conclusion:* The association and correlation between VN, MDA and hs-CRP indicate their involvement in the atherosclerosis process that may lead to progression of CAD. Also, these findings suggested that serum levels of VN, MDA and hs-CRP can help as diagnostic and monitoring markers in CAD patients and as markers of disease severity.

## Introduction

Coronary artery diseases (CAD) account for one of the major worldwide causes of morbidity and mortality. It is characterized by platelets activation and aggregation, thrombus formation and subsequently infarction.^1,2^ Different studies have suggested that at least 250 factors are related with the development of CAD.^3^ Molecules that have been identified newly and are associated with cardiovascular disease include those associated with impaired coagulation or fibrinolysis, cardiovascular remodeling and inflammation. High sensitivity-C reactive protein (hs-CRP), pro-oxidants, lipids such as malondialdehyde (MDA) and cell adhesion molecules including vitronectin (VN) are examples of such molecules.^3-5^ VN is found in plasma at concentrations of 200-400 μg/ml and constitute 0.2%-0.5% of total plasma proteins. Previous reports have shown that the liver may be the major source of plasma VN. In rodents, a high VN level has found in the liver. Also, high levels of VN mRNA were also detected in many other organs, including brain, heart, testes, thymus, skeletal muscles, lungs and uterus.^3,4^ VN is a major plasma protein which have found also in the granules-α of blood platelets and also in extracellular matrix of many tissues.^1,4^ VN binds to the multiple ligands, such as integrins, plasminogen activator inhibitor (PAI-1), urokinase plasminogen activator receptor (UPAR), complement-7, collagen, and heparin. These relations indicate that VN is implicated as a regulator of various physiological processes including peri-cellular proteolysis, coagulation, fibrinolysis, complement mediated immune responses, cell migration and attachment.^4,5^ Thus, VN serves a exceptional regulatory linkage between cell adhesion and physiological proteolysis.^5,6^

Numerous studies have mentioned that accumulation of free radicals has vital and causative role in the pathogenesis of atherosclerosis.^7,8^ Lipids especially low density lipoprotein (LDL) are many susceptible to be attacked by free radicals. Oxidative modified LDL (Ox-LDL) has a critical role in the evolution of the pathological state. Uptake of Ox-LDL by macrophages as well as smooth muscle cells causes the formation of foam cells, which is a significant step in the development of atherosclerosis. Oxidative activities of the free radicals are revealed by measuring their oxidative yields in biological systems.^9,10^ MDA is results from lipid peroxidation and its measurement is an undependable marker of oxidative damage. Therefore, MDA is a useful indicator and marker for identification and further assessment of patients with CAD.^11^

CRP is a hepatically derived pentraxin, composed of five 23kDa subunits, and has a critical role in innate immune response.^12^ Hs-CRP has known as a marker for low grade systemic chronic inflammation, and is directly involved in the endothelial dysfunction, platelet aggregation and atherosclerosis.^13^ This protein has been shown to have prognostic value in patients with acute coronary syndromes (ACS) and plays different roles in pathogenesis of atherosclerosis.^14^ Indicators of these phenomena such as hs-CRP, MDA and VN would be associated with the risk of CAD. Moreover, these biomarkers reflect different aspects of atherosclerosis progress in association with CAD risk.^15,16^

The study was conducted to assess the correlation of VN, MDA and hs-CRP with the severity of CAD. So, we prospectively tested whether parameters including VN, MDA and hs-CRP correlates together in these patients.

## Materials and Methods

### Subjects

A total of 250 individuals, 200 with CAD and 50 controls, were enrolled. CAD was diagnosed and evaluated by coronary angiography in Shahid Madani hospital of Tabriz University of Medical Sciences. Peripheral blood sampling was performed from CAD patients and normal peripheral blood samples were obtained from normal coronary angiography patients (controls) in Shahid Madani hospital. None of enrolled healthy subjects reported any past medical or family history of CAD. All patients with the history of any heart disease, lung disorder, liver dysfunction, renal disease and cancer were excluded from the study. Patient group comprised of 50 subjects with non-significant disease, 50 with single vessel disease (1VD), 50 with double vessel disease (2VD), and 50 subjects with triple vessel disease (3VD). The patients with non-significant disease had no obstructed vessels but suffered from chest pain like angina pectoris. The control group consisted of 50 subjects.

### Blood Sampling

Serum samples for measurement of VN, hs­-CRP and MDA were obtained from venous blood after a 12-hour fasting by centrifugation of clotted specimen within 30 minutes, and samples were kept frozen at −70°C until assays were carried out.

### Measurement of Parameters

Serum VN was measured using enzyme-linked immunosorbent assay (ELISA) procedure (Glory Science co. Ltd Cat. No: 11668). MDA was measured based on reaction with thiobarbituric acid (TBA), extraction with normal butanol, using the method suggested by Buege and Aust.^17^ Absorption measuring by spectrophotometer 535nm and were compared with standard curve. The serum hs-CRP was measured by high-sensitivity turbidimetry method using Biosystems kit (Barcelona Spain COD 31927); the assay was analyzed on semi-autoanalyser (Alcyon 300 made in USA) in the Biochemistry lab.^18^

### Statistical Analysis

**S**tatistical analysis was done using SPSS version 17. All variables were expressed as mean ± standard division. Differences among patient and control groups were analyzed using *t* test, Mann-Whitney U, and one way analysis of variance (ANOVA). Spearman coefficient was calculated to determine the correlation between biochemical parameters. *P* values less than 0.05 were considered significant.

## Results

The patient group consisted of 200 subjects with mean age of 58 years. The control group included of 50 subjects with mean age of 56.5 years (Table 1).

**Table 1:** The Demographic and Clinical Data of Patient and Control Groups

**Characteristic**	**Controls**	**CAD Patients**	*** P *** ** Value**
Number, n	50	200	-
Age (Mean±SD), y	56.5±8.4	58.0±9.8	.09
Gender (male/female), n	25/25	100/100	-
Smoker, n (%)	12 (24%)	110 (55%)	<.001
Diabetes, n (%)	7 (14%)	48 (24%)	<.001
Hypertension, n (%)	15 (30%)	118 (59%)	<.001

Comparison of mean values of VN in 1VD with normal and control groups showed significant difference, Also the mean values of VN showed significant difference between 2VD and 3VD groups (*P *= .03). VN in normal and control groups had significant difference with 3VD group (*P *= .001) (Table 2).

**Table 2:** Comparison of Mean Values of VN, MDA and hs-CRP Between Study Groups^a^

**Groups/ Parameters**	**Nonsignificant CAD**	**1VD**	**2VD**	**3VD**	**Control**	*** P *** ** Value**
VN	216.13 ± 49.59	375.88 ± 297.90	277.40 ± 123.74	400.74 ± 243.12	198.70 ± 46.47	<.001
MDA	5.41 ± 1.62	5.64 ± 1.47	6.54 ± 0.87	6.79 ± 1.79	4.82 ± 1.41	<.001
Hs-CRP	4.92 ± 5.70	5.42 ± 5.36	8.35 ± 5.96	5.81 ± 5.99	2.75 ± 3.45	.005

^a^ Data were showed as Mean ± SD.

The mean value of MDA between 1VD and 3VD groups showed significant difference (*P *= .01), Also mean value of MDA between 2VD with control and normal groups showed statistical significant difference (*P *< .001). The mean value of MDA between 3VD with normal and 1VD groups and control groups were significant (*P *< .001) (Table 2). The mean value of hs-CRP between 2VD and control groups showed significant difference (*P *= .003) (Table 2 and 3).

**Table 3:** Comparison of Mean Values of VN, MDA and hs-CRP in Study Groups^a^

**Parameters/Groups**	**Patient**	**Control**	*** P *** ** Value**
VN	347.74±231.10	198.70±46.47	<.001
MDA	6.35±1.48	4.82±1.41	<.001
Hs-CRP	6.57±0.55	2.75±0.77	.006

^a^ Data were showed as Mean ± SD.

Spearman correlation coefficient test showed that there was no statistical significant correlation between VN with MDA and hs-CRP also between MDA and hs-CRP in patient and control groups (Tables 4 and 5).

**Table 4:** Correlation Between MDA Value With Hs-CRP and VN

**Groups/Parameters**	**Hs-CRP**	**VN**
Patient	r = 0.2, *P *= .8	r = 0.2, *P *= .8
Control	r = 0.30, *P *= .18	r = 0.15, *P *= .51

**Table 5:** Correlation Between Serum VN With Hs-CRP

**Groups/Parameters**	**Hs-CRP**
Patient	r = 0.1, *P *= .34
Control	r = 0.1, *P *= .8

## Discussion

Atherosclerosis is considered as an intimal thickening due to complex interaction between endothelium and inflammatory cytokines.^19^ This pathological process includes monocyte, T-lymphocytes, and smooth muscle cells (SMCs) with addition and deposition of lipids and extracellular matrix proteins especially glycoprotein.^19,20^

Platelets are strictly related with elevated risk of cardiovascular events.^21^ They have integrins of the β_1_ and β_3_ subfamily, and platelets are activated following binding of integrins to adhesion molecules expressed by injured or inflamed endothelium. The activated platelet also expresses the integrins α_пb_β_3_ which causes the platelet to bind bivalent fibrinogen and subsequently crosslink with other platelets (aggregation).^22,23^ VN connects to platelet glycol proteins and mediates adhesion and aggregation of platelets at the sites of endothelial damages.^24^ Asch and Podack^25^ explained that anti-VN antibodies inhibit aggregation of platelets in vitro, indicating that VN plays role in platelet accumulation at the sites of endothelial injuries. However, Mohri and Ohkubo^26^ suggested that VN inhibits platelet aggregation and compets with Von Willebrand factor and fibrinogen for binding to glycoprotein IIb/IIIa receptor. This result revealed that VN prevents platelet-dependent thrombosis. In addition to its role in platelet interactions, VN controls the thrombotic response evoked by vascular injury by regulating thrombin function.^26^ Studies have shown that VN accumulated in human atherosclerotic plaques that are dependent on the VN receptor α_V_β_3_ and α_V_β_5_ play an important role of migration of SMCs into the intima layer which is a main contributor to intima thickening in atherosclerotic lesions.^27^ VN is involved in homeostasis, cell adhesion and migration and stabilization of PAI-1.^28^ VN binds PAI-1 and regulates its action by stabilizing the active PAI-1 conformation, and potentially regulates PAI-1 clearance.^29^ PAI-1 deficiency in human is associated with abnormal bleeding, indicating the crucial role of PAI-1 in stabilization of hemostatic clot.^30^ Elevated serum PAI-1 is associated with increased prevalence of ACSs such as deep venous thrombosis (DVT), unstable angina (UA), myocardial infarction (MI) and reinfarction, and sudden cardiac death (SCD).^31,32^ VN competes with heparin in binding to antithrombin-III, and consequently preventing the rapid inactivation of thrombin and factor Xa by this protease inhibitor.^6,33^ Derer et al^1^ suggested that VN is a clinically useful biomarker for unfavorable cardiovascular outcomes in patients with ACS undergoing coronary interventions.

Our findings are compatible with the previous reports^4,21^ and showed that serum VN in patients with CAD were elevated and correlate with disease severity.

Stoop et al^34^ reported that PAI-l/VN complex serves as a physiologic inhibitor of activated thrombin on the atherosclerotic endothelium. Moreover platelet activation, vessel wall injury, and increased expression of VN in endothelial and smooth muscle cells (SMCs) is contributed to high VN levels in serum of patients with CAD.

A lot of literatures have reported that free radicals are entrapped in cardiac ischemic injury. These free radicals are generated in the body during oxidation process. In normal condition they are removed by the diverse antioxidative defense systems found in blood and related tissues. During states such as myocardial ischemia, or ongoing MI, high levels of free radicals may be generated.^34^ MDA is produced from breakdown of lipids during peroxidation processes and serum MDA a reliable marker of oxidative damages. Pucheu et al^35^ suggest that serum MDA as a useful marker of oxidative stress during reperfusion of ischemic myocardium. Sharma et al^36^ studied young CAD patients and indicated a significant increasedin serum MDA levels compared to control groups. Dincić et al^37^ suggested that activity of free radicals in patients with myocardial ischemia is more increased than control individuals. Our results also indicated a significantly increased serum MDA, as a potent marker of lipid peroxidation in CAD patients compared to control groups, which correlated with disease severity.

Cigarette smoking which negatively affects the lipid profile also raise the production of oxygen free radicals. Wang et al^38^ studied smoker and nonsmoker healthy men and reported that serum oxygen free radicals are more increased in smokers than nonsmokers. We also studied smoker and nonsmoker patients with CAD, and revealed significantly increased serum MDA in smokers with CAD than nonsmoker CAD patients. So, elevated level of MDA in subjects may has a role in atherosclerosis, leading to increased cardiovascular risk.

A lot of evidences have indicated that inflammation has critical role in the development of CAD. Previous studies suggest that inflammation is an important provoking tool of coronary syndrome in the process of plaque rupture and directly and actively participate in promoter of atherosclerosis_._^12,39^ Majority of studies suggested that hs-CRP is cautiously sensitive systemic marker for diagnosis of inflammation and a useful and potent predictive marker of cardiovascular events.^2,36^ Pearson et al^40^ suggested that serum hs-CRP levels of <1, 1 to<3, and ≤3 mg/L is predictive of low, moderate, and high vascular risk, respectively. Our results are in accordance with the previous results.^2,11,12,36^ In our study, a significant difference was observed regarding the value of hs-CRP in CAD patients as compared to control groups, and there was an association between hs-CRP and CAD.

Our study indicated no relationship between VN, MDA and hs-CRP with together, hypertension and smokers. However, there was significantly high level of MDA in smokers with CAD than nonsmoker CAD patients.

## Conclusion

We suggest that serum levels of Vitronectin, MDA and hs-CRP increased in CAD subjects and potentially represent a pathogenic factor for atherosclerosis. Hence, we recommend use of these biomarkers as a diagnostic apparatus for CAD patients. However, more studies will be required to confirm this hypothesis.

## Ethical Issues

The ethics committee of Tabriz University of Medical Sciences approved the study. Informed consent was obtained from study patients.

## Competing Interests

Authors declare no conflict of interest in this study.
